# Qualitative Analysis of Emotions: Fear and Thrill

**DOI:** 10.3389/fpsyg.2016.01187

**Published:** 2016-08-10

**Authors:** Ralf C. Buckley

**Affiliations:** International Chair in Ecotourism Research, School of Environment, Griffith UniversityGold Coast, QLD, Australia

**Keywords:** risk, outdoor, emergency, sawtooth, autoethnography, emotionless

## Abstract

People can speak, and this provides opportunities to analyze human emotions using perceived experiences communicated via language, as well as through measurement and imaging techniques that are also applicable to other higher animal species. Here I compare four qualitative methodological approaches to test if, and how, thrill depends on fear. I use eight high-risk, high-skill, real-life outdoor adventure recreation activities to provide the test circumstances. I present data from: >4000 person-days of participant observation; interviews with 40 expert practitioners; retrospective autoethnography of 50 critical incidents over 4 decades; and experimental autoethnography of 60 events. Results from different methods are congruent, but different approaches yield different insights. The principal findings are as follows. Individuals differ in their fear and thrill responses. The same individual may have different responses on different occasions. Fear boosts performance, but panic causes paralysis. Anxiety or apprehension prior to a risky action or event differs from fear experienced during the event itself. The intensity of pre-event fear generally increases with the immediacy of risk to life, and time to contemplate that risk. Fear must be faced, assessed and overcome in order to act. Thrill can occur either during or after a high-risk event. Thrill can occur without fear, and fear without thrill. Below a lower threshold of perceived risk, thrill can occur without fear. Between a lower and upper threshold, thrill increases with fear. Beyond the upper threshold, thrill vanishes but fear remains. This there is a sawtooth relation between fear and thrill. Perceived danger generates intense focus and awareness. Fear and other emotions can disappear during intense concentration and focus. Under high risk, the usual emotional sequence is fear before the action or event, then focus during the action or event, then thrill, relief, or triumph afterward. The emotionless state persists only during the most intense concentration. For events long enough to differentiate time within the events, fear and thrill can arise and fade in different fine-scale sequences.

## Introduction

Neurological and endocrine study of human emotions has advanced greatly in recent years (LeDoux, 2012a,b; Lindquist et al., 2012; Adolphs, 2013). Neuroimaging techniques have been used to correlate emotions with neural signatures, and signatures with emotions (Vytal and Hamann, 2010; Baucom et al., 2012; Hamann, 2012; Lindquist and Barrett, 2012; Kassam et al., 2013; Kragel and LaBar, 2013; Woo et al., 2014; Chang et al., 2015; Howlett and Paulus, 2015).

Some aspects of emotional psychology, however, cannot yet be examined using laboratory based approaches. These aspects can only be studied through the qualitative analysis of experiences communicated via language. The value of data obtained via autobiographical memory has recently received explicit recognition (Harrison and Loui, 2014; Boccignone and Cordeschi, 2015; Buckley, 2015a; Gardner et al., 2015; Morin et al., 2015). There have been far fewer field tests or methodological advances using these approaches, however, than for approaches based on technological observations and measurements.

In particular, there are some emotional circumstances which are experienced only by a limited number of individuals, and which cannot be reproduced fully through virtual or other experimental means. For example, experimental studies cannot place participants under a real and immediate risk of death. Most experimental studies of anxiety under threat, for example, use only mild threats (Sussman et al., 2015), though some have employed intravenous administration of cholecystokinin to induce panic (de Montigny, 1989; Adolphs, 2013). Recent reviews (e.g., Adolphs, 2013) therefore emphasize the value of studying the conscious experience of emotions as well as the neurological and endocrine aspects.

Here, therefore, I present a comparison between four qualitative approaches in the study of emotions experienced by practitioners of outdoor recreation, during incidents which do involve real risk of immediate death, and which generate fear and thrill. Specifically, I compare findings from retrospective and experimental autoethnography (Buckley, 2015a) against those from participant observation and conventional ethnography (Silverman, 2010; Punch, 2014), which involve the recording of autobiographical memories of research subjects (Gardner et al., 2015).

Whilst many human emotions reflect feelings toward other people (Wilutzky, 2015; Michael, 2016), both fear and thrill are most commonly occasioned by an individual’s physical surroundings. Fear may also be occasioned by other persons perceived as threatening, and both fear and thrill may be moderated by the presence or actions of companions. This study, however, focuses on fear and thrill generated by voluntary but potentially dangerous activities carried out during adventure tourism and recreation.

Current understandings of fear and thrill are first reviewed, with particular reference to high-risk outdoor recreation. Many people deliberately take risks, and experience fear, in the quest for thrills. Each of the four qualitative methodological approaches is then presented, and each is compared against current understandings, to test whether it can yield new insights into: the factors influencing experiences of fear and thrill; the timing and trajectory of each under various circumstances; and the relationship between the two distinct emotions.

### Fear, Thrill, and Outdoor Recreation

From an experiential perspective, emotions have been analyzed within three different frameworks: as points in a multidimensional emotional continuum; as a set of >100 distinct named emotions in various groups; or as combinations of a small set of basic emotions, commonly recognizable from vocalizations and/or facial and bodily expressions (Johnson-Laird and Oatley, 1989; Meeren et al., 2005; Stets, 2006; Nesse and Ellsworth, 2009; Izard, 2010; Ebner et al., 2012; Graham and LaBar, 2012; Niedenthal and Brauer, 2012; Gu et al., 2013; Kliemann et al., 2013; Kret et al., 2013; Scherer and Meuleman, 2013; Lewinski, 2015).

Fear has an important place in each of these frameworks. Fear is routinely referred to as a basic or fundamental emotion. It is commonly recognizable from facial expressions. It includes a group of related terms such as anxiety, trepidation, apprehension, dread, fright, panic, and terror. The biochemistry and neurophysiology of fear are heavily studied (Chiao et al., 2008; Mujica-Parodi et al., 2009; Haegler et al., 2010; Adams et al., 2011; Agren et al., 2012; Adolphs, 2013; Avila and Lin, 2014; Dehaene, 2014; Zimmer and Clark, 2014; Sussman et al., 2015; White et al., 2016).

At a neurophysiological level, fear can arise through multiple pathways, and fear of pain is different from fear of attack (LeDoux, 2000, 2012a,b; Gross and Canteras, 2012; Adolphs, 2013; Tordjman et al., 2013). Humans also emit and detect fear pheromones (Ackerl et al., 2002; Mujica-Parodi et al., 2009; de Groot et al., 2015; Haviland-Jones et al., 2016). Individuals exposed to these, or to other cues of fear or threat, experience heightened perception and more rapid cognitive processing (Chen et al., 2006; Grillon and Charney, 2011; Grupe and Nitschke, 2013; Robinson et al., 2013; Stout et al., 2013; Jackson et al., 2014; de Valk et al., 2015; Sussman et al., 2015; White et al., 2016) and may take more risks (Maner and Gerend, 2007; Haegler et al., 2010), though this also depends on anger (Habib et al., 2015). When facial expressions of fear (Zhao et al., 2013) are ambiguous, humans also rely on chemical signals to interpret fear in others (Zhou and Chen, 2009; Graham and LaBar, 2012; de Groot et al., 2015).

Reported experiences of fear (Habib et al., 2015) have been described for people of different personalities and professions in various circumstances and situations, from children playing (Sandseter, 2009) to military activities (Fenz and Epstein, 1967; Caputo, 1977; Wallenius et al., 2004; Berko and Erez, 2005; Cohn et al., 2010; Taverniers et al., 2011; Bouchard et al., 2012; Lane et al., 2012; Johnson et al., 2014).

Psychological frameworks for human emotions treat thrill as less fundamental than fear. Related terms include excitement and exhilaration. Emotions such as joy, and triumph or exultation, may often be linked to thrill but are perceived as clearly distinct. There has been less biochemical and neurophysiological research for thrill or excitement than for fear. As with other emotions, thrill is influenced both by genetics (Dmitrieva et al., 2010; Thomson et al., 2013) and neurology (Zald et al., 2008; Kruschwitz et al., 2012). Suggestions of links between thrill and fear are longstanding (Kendrick, 1991; Mura, 2010) but previously untested. In valence-arousal classifications of emotions, fear is considered a negative emotion, and thrill positive. Such links would thus represent a transition from negative to positive valence.

Outdoor adventure recreation (Swarbrooke et al., 2003; Buckley, 2006, 2010a) provides opportunities to study powerful human emotions, including fear and thrill, in partially controllable settings (Celsi et al., 1993; Vermeir and Reynier, 2007; Allman et al., 2009; Brymer and Oades, 2009; Buckley, 2012, 2015a,b). Many types of adventure recreation, especially at expert level, involve significant physical risk. They require a high level of learned skill, rapid and precise coordination between sensory perceptions and bodily actions, and an ability to overcome fear. There are many ethnographic analyses of such activities (Michel et al., 1997; Brymer and Oades, 2009; Buckley, 2012), and several relevant neurophysiological studies (Pierson et al., 1999; Haegler et al., 2010; Taverniers et al., 2011; Van Westerloo et al., 2011). For guided commercial adventure tourism and recreation, guides choreograph and manage their clients’ emotional responses (Arnould and Price, 1993; Muloin, 1998; Holyfield, 1999; Montag et al., 2005; Sharpe, 2005; Pomfret, 2006; Curtin, 2010; Van Dijk et al., 2011; Wu and Liang, 2011; Buckley, 2012; Houge Mackenzie and Kerr, 2013).

Fear plays a key role in many adventure activities (Holyfield and Fine, 1997; Holyfield, 1999; Jonas, 1999; Beedie and Hudson, 2003; Vermeir and Reynier, 2007; Schnädelbach et al., 2008; Brymer and Oades, 2009; Carnicelli-Filho et al., 2010; Faullant et al., 2011; Buckley, 2012; Brymer and Schweitzer, 2013). This applies especially when fear must be mastered or managed (Fenz and Epstein, 1967; Arnould and Price, 1993; Slanger and Rudestam, 1997; Holyfield, 1999; Sharpe, 2005; Buckley, 2010b; Faullant et al., 2011; Houge Mackenzie and Kerr, 2013).

Thrill and exhilaration are reported by many adventure practitioners (Cater, 2006; Sicard et al., 2007; Tung and Ritchie, 2011; Buckley, 2012; Kim et al., 2012; Chen et al., 2013; Monasterio, 2013; Schlegelmilch and Ollenburg, 2013; Tsaur et al., 2013). High scores on the adventure and thrill-seeking subscale of the sensation-seeking personality scale (Zuckerman, 2007) have been reported, though not uniformly, for outdoor adventure activities including skydiving, paragliding, hang-gliding, bungy jumping, rodeo riding, skiing, surfing, and whitewater kayaking (Straub, 1982; Rainey et al., 1992; Campbell et al., 1993; Chirivella and Martinez, 1994; Wagner and Houlihan, 1994; Michel et al., 1997; Zarevski et al., 1998; Gomà-i-Freixanet, 1999; Pierson et al., 1999; Franques et al., 2003; Diehm and Armatas, 2004; Zuckerman, 2007, pp. 97-100; Barbieri and Sotomayor, 2013). For skilled outdoor sport, recreation and adventure, there are ethnographic and autoethnographic analyses of emotions such as fear and thrill in a wide range of activities (Holyfield, 1999; Houge Mackenzie and Kerr, 2013; Buckley, 2014).

No previous formal studies, however, by any method, have set out specifically to examine links between fear and thrill. Analyzing such links is not straightforward. These are human emotions, experienced only by the individuals concerned, often only briefly and under conditions of considerable stress. They are not directly quantifiable by observers. They are not recorded except in individual memories, and indirectly through recorded observations of any outward expressions. Here I report a robust approach that compares four independent sources of data, from different individuals at different times and places: a quadrangulation of qualitative methods.

## Materials and Methods

### Ethics Statement

Some of the data presented here were obtained during commercial adventure tours. Assistance and sponsorship of tour operators is gratefully acknowledged. All quotations from individual interviews are used here with the informed written consent of those concerned. All of the research reported here, including that drawn from previous publications by the same author (Buckley, 2006, 2007, 2010a,b, 2012, 2014, 2015a,b), as well as new research conducted specifically for the current publication, was conducted in strict compliance with the research ethics requirements of Griffith University, including adherence to the Griffith University Research Ethics Manual and the Australian National Statement on Ethical Conduct in Human Research, and approvals by the Griffith University Human Research Ethics Committee as required.

### Quadrangulation

Four different methods were compared, a quadrangulation of approaches. These were: participant observation, practitioner interviews, retrospective autoethnography, and experimental autoethnography. All four used voluntary participants in outdoor recreation and adventure tourism. Participant observation, practitioner interviews, and retrospective autoethnography are established methods (Spradley, 1980; Dewalt and Dewalt, 2002; Buzard, 2003; Anderson, 2006; Dauphinee, 2010; Silverman, 2010; Tolich, 2010; Denzin, 2014; Punch, 2014; Buckley, 2015a). Experimental autoethnography is a recently proposed method (Buckley, 2015a), used here for the first time.

The four methods were applied for similar outdoor adventure activities, but over different time periods, in different places, with different subjects. For participant observation, practitioner interviews, and retrospective autoethnography, multiple activities were studied: surfing, sailboarding, and kiteboarding, helicopter snowboarding, whitewater kayaking and rafting, hang-gliding, climbing and mountaineering, off-road driving in remote areas, and watching large and potentially dangerous wildlife. For experimental autoethnography, only two activities were examined, namely surfing and kiteboarding. Each approach provides a different combination of breadth and depth. Results were compared to provide reliability and additional insight.

All observations, document and video analyses, and interviews were conducted and analyzed in accordance with institutional research ethics requirements and national research ethics rules (see Ethics Statement), and with international best practice, including those for interviews, ethnographic, and autoethnographic approaches (Silverman, 2010; Tolich, 2010). Interviewees were informed of the purpose of the research and the identity of the researcher, gave prior informed consent to take part, and gave written permission for individual quotes to be used. Anonymity of all interviewees and participants has been strictly preserved, even when individual participants gave permission to be identified. Some of the data used here were compiled during previously published research (Buckley, 2006, 2007, 2010b, 2012, 2014, 2015a,b), and the re-use and re-analysis of the data also complies with institutional ethics requirements (see Ethics Statement).

### Participant Observation

Participant observation data were compiled from >4,000 person-days’ activities over a period of a decade. Participants ranged in experience from novices to longstanding international guides and instructors. They represent the particular population of people who choose, and are able, to take part in adventure activities. This group is studied here specifically to provide information on links between fear and thrill. Actions, vocalizations and facial expressions were observed directly, and also recorded using multiple hand-held and head-mounted video cameras. As outlined earlier, both fear and thrill are commonly recognizable through facial expressions and vocalizations (Chen et al., 2011; Van den Stock and de Gelder, 2014; Martinez et al., 2015), but these outward expressions can sometimes be suppressed (Gross and Barrett, 2011). Analysis of observations and recordings includes conversations where participants reveal or refer to emotions, which may not have been apparent from actions and expressions alone. Emotions recognized in the field from facial expressions and vocalizations, were necessarily classified only by the author, or by the author and participant where conversations were also involved. Where video recordings were made, emotions were recognized jointly by the entire participant group during playbacks on the same day or at the end of the trip: effectively, a focus-group approach to cross-coding.

### Practitioner Interviews

Expert practitioner interviews were derived ethnographically from semi-structured face-to-face and electronic interviews with skilled guides, instructors and practitioners, over a 3-year period. These individuals have carried out adventure activities over many years, often involving high danger and high risk of immediate death. No inducements were used; interviewees were informed of the research context; procedures followed requirements for research rigor and ethics (Tong et al., 2007; Dauphinee, 2010; Tolich, 2010); and standard emic qualitative analysis approaches were used (Silverman, 2010; Punch, 2014). Ethnographers can use their own experiences, shared with others, to interpret those others’ experiences (Hammersley and Atkinson, 2007), because emotional experiences are comprehensible between individuals (Johnson-Laird and Oatley, 1989; Izard, 2010). Individuals reporting similar emotional experiences exhibit similar changes in brain biochemistry and electrical activation, and similar though culturally mediated facial expressions and bodily postures and gestures; and brain parameters and reported emotions respond consistently to experimental manipulation (Frank and Temple, 2009; Niedenthal and Brauer, 2012; Dehaene, 2014). Despite these strong similarities, however, individual sensory and emotional experiences and responses are not identical (Johnson-Laird and Oatley, 1989; Dmitrieva et al., 2010; Gross and Canteras, 2012; Bushdid et al., 2014).

### Retrospective Autoethnography

Retrospective autoethnographic analyses are based on 50 individual incidents experienced over a period of four decades, each involving fear, thrill and related emotions. Each recollection was written out factually at length, reconsidered and mentally replayed multiple times, and examined and compared iteratively, focussing on intensity and timing of fear and thrill. Retrospective analyses use memories, voluntary or involuntary (Berntsen, 2002, 2009; Rubin, 2014). Memories of highly emotional events, also known as critical incidents (Flanagan, 1954) remain intense and detailed for decades, and recollecting the events can re-create the original emotions (Mattley, 2002; Levine and Pizarro, 2004), even when the brain was not focused on the emotions at the time (Avila and Lin, 2014; Dehaene, 2014). Such memories record only specific brief events from a lifetime of experience, but these are precisely the events needed to study high-intensity emotions. Outdoor recreation practitioners have historically claimed that the intense emotions experienced during high-risk, high-skill outdoor recreation are ineffable, impossible to communicate to non-practitioners (Allman et al., 2009; Brymer and Oades, 2009). Recent approaches, however, have shown that they can be analyzed using retrospective autoethnography (Buckley, 2012, 2015a).

### Experimental Autoethnography

Experimental autoethnography involves deliberately repeating the same actions, and observing one’s own emotions as they are experienced (Buckley, 2015a). The observer is still reliant on memory to record the emotions experienced, but it is short rather than long-term, and it is cued by an external trigger rather than an emotional threshold. I used two actions to test the relation between fear and thrill: catching a steep fast wave on a surfboard, and jumping a kiteboard in surf and high wind. Each of these requires learned skills to undertake the maneuvre successfully; and in each case, failure or insufficient skill leads to a wipe-out, a loss of control followed by a fall. In large waves or strong winds, wipeouts can be violent and may cause injury or worse (Buckley, 2012). Each of these maneuvres involves quick, precise, and carefully timed judgements and actions, adjusted through sensory feedback and cognitive control, at subsecond timescales (Buckley, 2012). To track one’s emotions during these processes involves switching one’s attention, for very brief intervals, from observing action to observing emotion. This technique is not automatic, and has to be learned gradually during multiple repetitions of the same action.

## Results

### Participant Observation

Participant observations were compiled from >4,000 person-days activities over a period of a decade. These included: 273 days, >300 separate individuals, and >3000 participant-days of moderate to high-risk white-water rafting and kayaking, much of it in remote and unknown areas; 83 days, >90 individuals, and >440 participant-days of wildlife watching in potentially dangerous circumstances, such as watching bear, rhino, elephant, buffalo, lion, leopard, or tiger at close or very close range, on foot or in open vehicles, by night as well as day; 72 days, 46 individuals, and 365 participant-days of high-risk surfing on shallow tropical reef breaks; and 70 days, 55 individuals, and 350 participant-days of helicopter snowboarding, including steep, densely tree-covered, and heavily cliffed terrain with high avalanche risk at altitudes to 5000 m.

Participant observations provide a relatively coarse understanding of individual emotional experiences. More experienced participants rarely showed fear, even though they freely showed exhilaration and other strong emotions, e.g., when concerned for others’ safety. Less experienced participants showed frequent thrill and fear and occasional panic, as revealed by behavior, expressions and vocalizations. Highest fear was shown by first-time participants during unfamiliar high-risk activities. Where the same individuals were observed repeatedly, either on successive days of multi-day activities, or in multiple trips over successive years, incidence of panic decreased as experience increased.

The key result from this approach is that different individuals experience different degrees of fear and thrill, and the same individual may experience different fear and thrill during different events. Participants generally displayed fear or anxiety before a potentially risky event, focus within the event, and thrill afterward. They showed no fear after the event, only relief or triumph; no thrill before it, only anticipation; but sometimes, both fear and thrill during the event itself. Fine-scale sequences of fear and thrill during the event were generally not discernible using this method. That is, it did not prove possible to resolve, using participant observation alone, whether or not fear increases thrill.

### Practitioner Interviews

Individual interview data were derived ethnographically from semi-structured face-to-face and electronic interviews during 2012-2016 with 40 skilled guides, instructors and practitioners: rafters, kayakers, mountaineers, rock and ice climbers, surfers, sailboarders, kiteboarders, and snowboarders. Except for mountaineering and climbing, I had sufficient expertise in each of these activities to be accepted as a competent intermediate practitioner, able to exchange technical and emotional experiences with interviewees. However, my skills have always been well below those of the interviewees themselves. These expert practitioner interviews yielded seven main results, illustrated by the quotations below. The views expressed during these interviews are also reflected in quotations from expert practitioners published in the popular literature of adventure recreation (Davis, 2012; Moriarity, 2012; Yogis, 2013).

(1) For most individuals, fear increases thrill, but only up to a point. *“If there’s no fear, the achievement is not nearly as fulfilling.” “Fear makes the thrills better.. take on being scared and try and conquer it.” “The thrill of being scared... the real thrill is conquering the fear.” “Fear definitely adds to the enjoyment … a feeling of accomplishment.” “Fear increases thrill sometimes, but not always.” “Fear does increase thrill, but there is a fine line between excitement and terror”.* (2) Only one informant disagreed, a polar explorer who experiences months rather than moments of risk: *“I get no thrill from fear.” “Moments of fear … add no pleasure.”*

(3) Fear before an activity, anxiety, is different from fear during an activity, which may include panic. *“Fear.. in anticipation..” “there’s pre-adventure fear and ‘in the moment’ fear…” “Fear can hit suddenly.. like ‘I could die here’.”* (4) Fear must be faced, assessed and overcome. *“Stay centred … lay the fear that you have in the palm of one hand and the risk that you face in the other, don’t let go of either.” “Fear.. is an alert system for my judgement process.” “Overcoming fear so as to act.”* Panic or terror are experienced as extreme fear which clouds judgment and blocks action. The ability to avoid panic and overcome fear increases with skill and experience.

(5) Fear can disappear during intense concentration. *“Fear increases focus and concentration.” “During the activity, concentration is so intense that fear drops off.” “During the run there’s no time for fear.”* (6) Thrill sometimes occurs after fear, but not always. *“Fear comes before the activity. Thrill comes at the end.. a mixture of relief, exultation, satisfaction and adrenaline.” “Thrill comes, once I’m in it and once I’ve completed it.”* (7) For some interviewees, fear itself is not enjoyable, but thrill is. *“Fear itself is not at all pleasurable … but moving beyond the shackles of fear … is deeply rewarding.”*

The key result from this approach is that for most practitioners, fear increases thrill, but only up to a point. Expert practitioners agree that: fear must be faced, assessed and overcome; fear can disappear during intense concentration; and fear is often, but not always, followed by thrill. They differentiate: fear before an activity starts from fear once the activity is under way; fear from risks beyond one’s control, from fear associated with one’s own capability and performance; and manageable fear, which improves focus, from unmanageable panic, which induces paralysis. The paralyzing consequences of panic match those reported by Adolphs (2013).

### Retrospective Autoethnography

This approach was used here to examine emotions experienced during infrequent dangerous incidents in high-skill high-risk outdoor recreation activities. The activities were: hang-gliding, whitewater kayaking, surfing, off-road driving, snowboarding, surfing, and wildlife watching. Most of the time, these activities produce thrills with little or no risk or fear, and are not recalled as critical incidents. The data for this approach comprise 50 individual incidents, over four decades, which did involve substantial fear, and sometimes also thrill and other powerful emotions. Most incidents lasted only minutes, seconds, or less. A selection of the more extreme incidents are summarized below. Some of these incidents occurred during individual private recreation, others during guided commercial adventure tours. At the time when each incident took place, my experience qualified me in the higher categories of recognized expertise, such as Class V whitewater and equivalent levels for hang-gliding and snowboarding. However, my expertise has always been far below that of extreme or internationally competitive athletes in any of these activities.

Nine of the incidents involved hang-gliding. In two, I witnessed fatal crashes during cliff take-offs, and then flew off the same cliffs myself. This involved high fear, extreme concentration and intense awareness, overriding thrill. Four involved unusual, unanticipated and dangerous circumstances. In one, an error in harness attachment caused over 100 involuntary stalls in 10 min, before I could reach the ground. In another incident, I was hit by a powerful storm front lasting 15 min, and crawled into the nose wires of the hang-glider to maintain tenuous control. Under normal flight conditions this would cause a nosedive and crash. In a third incident, I crashed into a 30m tree and fell out backward and upside down, still attached to the hang-glider. In a fourth, a military jet flew beneath my hang-glider, at supersonic speed. There was no thrill in any of these, but very intense awareness. A number of incidents involved powerful thrill as well as fear. These include: rapid height gain in very turbulent thermal lift; semi-inverted acrobatic maneuvres at the limits of the hang-glider’s flight capabilities; and once, two large eagles flying below my wingtip.

Nine incidents involved surfing. Surfing large steep waves breaking violently over shallow seafloor involves substantial risk of injury, concussion or drowning, especially in crowded conditions over coral reefs. Apprehension and continuous cognitive reappraisal of risk (Dunsmoor et al., 2012) is commonplace whilst paddling into position. The moment of commitment to take-off can generate “heart in mouth” fear, but the actual take-off, which may involve balanced free-fall down the wave face, involves very intense concentration, overriding all emotions. Successful take-offs onto steep fast waves, either long open walls or hollow barrels, yield thrill or joy (Buckley, 2012; Hosany, 2012). Fear and thrill may often be felt on the same wave.

Seven incidents involved backcountry snowboarding, including heliboarding. In one, I triggered a large slab avalanche, and barely escaped by riding at speed over the buckling and cracking slab, and jumping from moving to stationary snow at the edge. There was an instant of fear followed by intense concentration. In another, I made an emergency stop in soft snow, 20 cm from a 30-m vertical cliff onto rocks. Recalling this still produces a cold sweat. There was no thrill in either of these incidents. Intentional jumps onto steep snow slopes, in contrast, produce high thrill as well as fear. The same applies to high-speed descents down narrow snow-filled couloirs with high avalanche risk, or through dense trees on deep dry powder snow, with risk from tree-wells or tree collisions. There were also many occasions that generated thrill without fear, e.g., riding large open bowls or steep faces in deep dry powder snow with low avalanche risk.

Fifteen incidents involved white-water kayaking through river rapids, waves, and complex turbulent hydraulic features studded with rocks. Most of the time this generates high thrill and low fear. Features such as whirlpools, waterfalls, and more difficult and extended rapids generate fear beforehand, intense concentration during the maneuvre, and high thrill afterward. Paddling rapids that have never been run before (Van Beek, 1998) or where other kayakers have drowned, generates additional fear. Some incidents generated fear without thrill, notably several where I was trapped under water for extended periods. Running long complex rapids, one has time to become aware of both fear and thrill. Other manoeuvres, such as kayak-surfing large standing river waves with low risk, generate thrill without fear. They may be broadly memorable, but are not recalled in high detail as critical incidents.

Seven incidents involved off-road travel in four-wheel-drive vehicles, days away from any other people, or any chance of rescue. In one, the wheels cut through the crust of a desert salt lake, and I barely escaped. There was a moment of fear, followed by intense emotionless concentration. In another, I became lost in a desert flooded by cyclonic rains. I felt not fear, but exhaustion and determination. When I eventually escaped, I was overcome by powerful and incoherent relief. Other incidents involved driving through windblown dunes with unstable slip-faces and sand-traps, or deep river crossings with water up to the windows of the 4WD. These involved trepidation before starting, intense concentration during the event, and relief and triumph on completion.

Thirteen incidents involved close encounters with large or potentially aggressive animals, either marine or terrestrial. Two humpback whales swam in circles, at close range and high speed, around my unstable racing surfski, several kilometers offshore. This generated thrill but also trepidation. Diving with unaggressive shark species in shallow water, or swimming with seals and sealions, generates thrill rather than fear. Diving with hammerhead sharks at depth or at night, however, does generate fear. Several times I have been within arm’s length of lion or elephant, either in an open vehicle or on foot, sometimes at night. This involves very close attention to the animal’s behavior, and a certain degree of both fear and thrill. I have been attacked by a baboon, and bitten by a troop of monkeys. On one occasion I and two companions on foot were charged at short range by a black panther, a melanistic leopard. On that occasion I felt no fear but very rapid mental processing.

The principal results from this approach may be summarized as follows. From a methodological perspective, memories are most intense and detailed for incidents involving powerful fear, and the selection of incidents necessarily reflects that. Incidents involving thrill more than fear were far more numerous, but are not recalled so specifically. Acknowledging this intrinsic selectivity, these 50 critical incidents support the conclusions from the expert practitioner interviews, and add further detail. Thrill can be experienced without fear, and fear without thrill. Where both are experienced during the same incident, then fear increases thrill up to a certain threshold, but beyond that, thrill disappears. During brief intense incidents where perceived risk is high and immediate, there is a sequence of emotions or perceptions: fear before the event, emotionless focussed concentration during the event; and thrill, relief, and/or triumph after the event. During more extended incidents, lasting seconds, or minutes, fear can reappear during the event, and fear and thrill may alternate in response to very short-term actions and perceptions.

### Experimental Autoethnography

Both surfing and kiteboarding depend on ambient wind and wave conditions, so they can only be carried out when conditions are appropriate. In addition, only a very small proportion of the time spent surfing and kiteboarding involves take-offs and jumps, respectively. Most of a surfing or kiteboard session, which may typically last one or more hours, is spent paddling into position or sailing back and forth. Each individual surf take-off or kite jump typically takes a few seconds at most, often less for surf take-offs, and rarely up to 15 s for a large jump. For both surf take-offs and kite jumps, control is needed throughout the maneuvre. In each case, however, the crux component is shorter still, and requires precise perceptions and physical movements at timescales of a 10th of a second. This approach thus generates only a few seconds of data from many hours of activity over an elapsed period of more than 2 years. There are no absolute thresholds of perceived risk, fear, or thrill, distinguishing surf take-offs or kiteboard jumps which provide data, from those which do not. There is thus no absolute count of the number of incidents. There were at least 30 incidents in each activity, however, where both fear and thrill were distinctly recognizable.

Results from experimental autoethnography support those from expert practitioner interviews and retrospective autoethnography, and provide additional insights at finer timescales. In contrast to retrospective autoethnography, the experimental approach is intrinsically biased toward events with lower risk and hence lower fear, since the manoeuvres are deliberate and the author is not an extreme outdoor athlete. In this approach, fear was low, thrill was high, and there were no incidents involving fear without thrill. This approach also supports the sawtooth relation between fear and thrill, where fear boosts thrill up to a certain threshold but not beyond. Whilst many of the incidents used in retrospective autoethnography, however, were beyond that upper threshold, all of the incidents in experimental authoethnography were well below it. The experimental approach also shows that the sawtooth threshold can differ with conditions, for the same activity and individual.

Experimental autoethnography showed that different combi nations or categories of fear and thrill may be distinguished. This is consistent with findings from other approaches, but more detailed. On days with a large surf or a strong wind, such as during cyclonic conditions, there is a generalized pre-event anxiety and excitement in deciding whether to enter the water at all, and in getting ready to do so. This is essentially a recognition of risk related to ambient conditions, which one judges carefully, assesses against one’s own capabilities, and overcomes by paddling out or rigging up. Once one is in the water, paddling the surfboard or riding the kiteboard, there is a very careful attention to conditions, which may include apprehension as well as awareness.

The most significant result from experimental autoethno graphy is that there are different short-term sequences of emotions, depending on actual outcomes and cognitive sense of control. This confirms analogous suggestions from practitioner interviews and retrospective autoethnography. As one starts the surf take-off or kite jump, the focus is entirely on control, and all emotions disappear. If the maneuver is successful, then this absence of emotion lasts for as long as powerful concentration is needed, throughout the entire maneuver and any subsequent components such as riding a fast and difficult section of a wave, or flying the kite through a jump and landing. Once this is completed, then emotions flood back in a powerful surge of thrill and triumph, which may not be felt until several seconds after the maneuver itself. If the latter part of the wave is easier to ride, or the kite jump involves a smooth maneuver lasting several seconds or longer, however, then one can feel thrill whilst still on the wave or in the air. This corresponds to results from retrospective analytical autoethnography involving snowboarding on long steep slopes, or whitewater kayaking on long rapids. The key finding is that the emotionless state persists as long as full concentration on controlled action is required.

If the maneuver is failing, however, then a powerful burst of “in-the-moment” fear can return instantaneously, overcoming the emotionless sensation. That is, if fear returns because of incipient failure, it does so much faster than thrill is felt following success. This burst of fear, however, is commonly short. Either it vanishes, if one regains control; or it is replaced by concentration on escaping danger and minimizing damage, if one does not. This corresponds to the findings of Noyes and Kletti (1972) that falling climbers “acted with lightning-quickness in accord with accurate judgment”. In such cases, there is no subsequent thrill, but only relief once one is out of immediate danger. It also corresponds to the findings from retrospective analytical autoethnography, as above, for the life-threatening incidents related to hang-gliding, snowboarding and whitewater kayaking. This result was not available from participant observation or expert practitioner interviews.

### Comparisons

Each of these four methods and datasets demonstrate links between fear and thrill under risk, but each reveals difference insights and nuances in that relation. The principal results from each approach are summarized in **Table 1**. Considered jointly, these four approaches yield two central conclusions, which do not seem to have been reported previously.

**Table 1:** Comparison of results from four qualitative methods in analyzing emotions.

Methodological approach	Participant observation	Practitioner interviews	Retrospective autoethno	Experimental autoethno
**Scope and data**				
Activities	4	8	7	2
Years	10	3	40	2
Scale	>4000 person-days	38 expert practitioners	50 critical incidents	60 incidents triggered
**Principal findings**				
Individuals differ in fear and thrill responses	*	*		
Same individual may have different responses on different occasions	*	*		
Three measures of past emotional intensity correlate only weakly			*	
Fear boosts performance, panic paralyses	*	*		
Pre-event fear differs from fear during event		*	*	*
Fear increases with immediacy of risk, and time to contemplate it			*	
Fear must be faced, assessed, and overcome in order to act	*	*	*	*
Thrill can occur during and/or after event	*	*	*	*
Thrill can occur without fear, and fear without thrill		*	*	*
Below a lower threshold, thrill can occur without fear	*	*	*	*
Between a lower and upper threshold, thrill increases with fear		*	*	
Beyond the upper threshold, thrill vanishes but fear remains			*	
Perceived danger generates intense focus and awareness		*	*	*
Fear can disappear during intense concentration and focus		*	*	*
Absence of emotion extends only during most intense concentration			*	*
Under high risk, emotional sequence is fear, focus, thrill/relief/triumph		*	*	*

* Finding demonstrated by method concerned.

Firstly, the relation between fear and thrill has a sawtooth format. If fear is low, it is possible to experience thrill without fear; and if fear is high, it is possible to experience fear without thrill; but within a certain range of intensity of fear, increased fear leads to increased thrill. As fear increases beyond an upper threshold, fear overwhelms thrill, thrill falls suddenly to zero, and only fear is experienced. The sawtooth threshold may occur at different fear intensities for different people, activities and circumstances. This sawtooth relationship has apparently not been identified previously.

Secondly, during brief intense incidents where perceived risk is high and immediate, there is a sequence of emotions or perceptions: fear before the event, emotionless focussed concentration during the event; and thrill, relief and/or triumph after the event. During more extended incidents, lasting seconds, or minutes, fear can reappear during the event, and fear and thrill may alternate in response to very short-term actions and perceptions. If so, the fear felt during the event is perceived as qualitatively different from fear felt before starting.

There are several apparently novel conclusions derived specifically from retrospective autoethnography. The first result relates to a researcher’s ability to recall their own emotions. There are three different measures of the intensity of emotion experienced during a past event (Mattley, 2002; Bywaters et al., 2004; Levine and Pizarro, 2004). The first measure is the remembered intensity of any involuntary physiological response that occurred during the past event. Involuntary physiological responses to fear include sensations perceived as big-eyes, prickly-skin or falling-stomach. Involuntary responses indicating other emotions include yells of excitement, roars of triumph, or gasps of relief. For example, in one of the off-road driving events, when I finally found a way out after being stuck in the desert, I recall leaping from the vehicle and roaring incoherently and involuntarily at the top of my voice. That is, I recall a very powerful emotion, but the process of remembering it is free of emotion.

The second measure of past emotion is the level of very fine detail in the memory, indicating heightened awareness during the event. For example, for one of the hang-glider cliff take-offs shortly after another participant had been killed, I have a very intense memory of sun on wildflowers, as I strove to overcome fear before hooking up my harness. The third measure of past emotion is the degree to which involuntary physiological responses are created by the process of recollecting the event. For example, when I recall nearly snowboarding off a cliff onto rocks, the recollection itself produces a cold sweat, though I have no memory of any such response at the time. The analytical autoethnographic approach thus indicates that for the author, the three measures of the intensity of past emotions are not well correlated. If this also applies more generally, it affects all conventional ethnographic studies of emotion.

The second new result from retrospective analytical autoethnography is that the degree of fear is driven by three factors. The first factor is the perceived probability of immediate death. Almost riding a snowboard off a high rock-strewn cliff is an example. The second factor is a longer period to contemplate death before a critical action, even if the risk during that period is low. An example is the period before a hang-glider cliff take-off, or before paddling over a waterfall or into a Class V whitewater rapid. Sometimes both these factors can occur simultaneously, for example when my hang-glider harness was incorrectly attached and I suffered an extended series of stalls, each with the risk of death. The third factor is that fear can be blocked or overridden by intense concentration on actions to avoid immediate death. This occurred during a number of these incidents, including being trapped underwater whilst surfing or kayaking, or driving a 4WD across a remote arid salt lake. The third of these factors is also supported by expert practitioner interviews, but that approach did not provide data on the first two. Now that these factors have been identified, it would be possible to test them more broadly through new expert interviews, as suggested by Buckley (2015a).

The third result from retrospective analytical autoethnography is that there are identifiable sequences of emotions, differing between more and less intense events. For activities with an extended series of moves, fear can intrude suddenly on thrill, depending on cognitive sense of control. Examples include: multiple manoeuvres in a long and complex whitewater rapid; high-speed linked snowboard turns around close-set trees on a steep powder slope; or a long sailboard or kiteboard ride on a large steep wave breaking fast. This fear is of the in-the-moment type. If one regains control, fear disappears again, leaving only a brief aftermath. This matches recent neurological findings (Agren et al., 2012). When the threat of death or other powerful fear is successfully overcome, fear gives way to relief or triumph. Again, this could be tested more broadly through new expert practitioner interviews.

Retrospective and experimental approaches to analytical autoethnography produce different insights. Since the incidents generating highest fear involved high perceived risk, and I have not experienced such incidents deliberately, they are only available for retrospective analysis. Experimental autoethnographic approaches with an external-cue trigger (Buckley, 2015a), in contrast, involve greater focus on thrill, at much lower intensities of risk and fear. This approach provides more reliable and detailed data on changes between emotions over very short time-scales. In addition, the experimental approach can provide successive sets of increasingly focussed observations.

It is difficult for an individual to recall, or describe in a later interview, exactly what sequence of emotions they experienced during a move or manoeuver which is at once difficult, dangerous, exciting, but frightening. In recollection, the various emotions may seem to have been mixed or confused, or to have followed each other at such short timescales that one cannot be sure of the order. It is also difficult for an observer to detect such a sequence, because it occurs too rapidly and may be revealed only weakly through facial expressions, especially if the observer cannot get very close to the person undergoing the experience. By repeating and remembering such experiences oneself, however, and deliberately paying attention to the emotions involved, it is possible to distinguish such a sequence. This is the key benefit of experimental analytical autoethnography in the analysis of emotions (Buckley, 2015a).

Fear and thrill are both extensively studied. The approaches used here add: a distinction between pre-event fear and in-the-moment fear; a distinction between empowering fear and paralyzing panic; and a sawtooth relationship between fear and thrill, with upper and lower thresholds which differ between individuals. Analytical autoethnography, both retrospective and experimental, shows that during high-risk, high-skill activities, fear, and thrill may occur jointly or succeed each other at very short timescales, and that there are multiple different pathways or trajectories, depending on timing, physical circumstances, and perceived control. No other approach has revealed this level of detail.

## Discussion

From the practical perspective, the sequence and drivers of emotions during outdoor adventure recreation are especially valuable if they can be used to analyze and improve emotional control during emergency services and military combat, where it is a great deal more difficult to conduct research. The expert ethnography, in particular, indicates that through practice, one can learn to avoid panic so as to think and act rapidly, decisively, and appropriately under high risk. It appears, though this requires further testing, that this approach only succeeds if the danger and fear are real (cf. Morreall, 1993).

Currently, emergency services and military personnel achieve this by practicing their skills during high-risk events in their actual professions, so-called combat hardening (Fenz and Epstein, 1967; Friedland and Keinan, 1992; Taverniers et al., 2011; Foster and Fletcher, 2013). To reduce injury and mortality, military and emergency services organizations use training approaches which simulate combat or emergencies (Cohn et al., 2010; Bouchard et al., 2012; Johnson et al., 2014). These, however, generally do not involve real fear. The results presented here suggest that it would be more effective to combine such training with practice in overcoming real fear, e.g., through adventure training. Many such organizations do engage in adventure recreation, but not yet for this purpose.

From a research perspective, three significant new avenues arise from the results reported here. The first is that they may help to elucidate how humans perceive the passage of time (Arstila, 2012; Wittmann, 2013; Buckley, 2014; Li and Yuen, 2015). The second is that, especially if it could be coupled with biochemical, neurological and musculoskeletal observations, analytical autoethnography could help to elucidate how we learn physical skills which require very fine coordination of observation and control. There are now mobile electroencephalographic devices such as those manufactured by Emotiv^®^ (2016), but to date these cannot be used in water, especially salt water. I look forward to the day when surf and kite helmets can be fitted with EEGs. Meanwhile, perhaps such a helmet could be constructed for use while snowboarding or bungy jumping.

The third question is: what is the evolutionary origin of thrill? Fear in the face of danger has clear ecological and evolutionary advantages (Nesse and Ellsworth, 2009; Adolphs, 2013; de Valk et al., 2015; Lu et al., 2015). But what is gained through thrill? Thrill drives individuals to perform actions that involve risk and fear. Why should this provide an evolutionary advantage? Is it, perhaps, a mechanism to induce juvenile humans, and perhaps other animal species, to practice the skills they need to survive, thrive, and reproduce in adult life? Humans experience both fear and thrill from a young age (Sandseter, 2009). It appears that as skill increases, the degree of risk and fear needed to generate the same level of thrill also increases (Buckley, 2012). It also appears that when thrill is coupled with the successful performance of a skilled activity under risk, generating what Buckley (2012) referred to as rush, this becomes addictive. These would both be consistent with a strong role in learning.

Similarly, it seems that when circumstances require maximum attention and concentration because of high risk, neither fear nor thrill nor any other emotion is experienced at that moment. This is not shock, because it is accompanied either by very rapid mental processing, or complete calm. If emotions have evolved to increase individual survival, why would they disappear at moments of highest risk? Could it be, perhaps, that biochemical aspects of emotion are valuable in long-term behavioral conditioning, but that they interfere with short-term neurological processing, so that when high-speed logical thought is critical for survival, emotions are temporarily shut down? This might be analogous to the recently described mechanism showing interferences between memory and stress (Sekine et al., 2015). More generally, perhaps high-risk adventure activities could contribute to understanding of links between emotion and cognition (Okon-Singer et al., 2015) if we could monitor very short-term changes in brain biochemistry and neurophysiology as individuals undertake activities with high risk, skill, fear and thrill.

## Author Contributions

RB conceived, designed and conducted research, wrote article.

## Conflict of Interest Statement

The author declares that the research was conducted in the absence of any commercial or financial relationships that could be construed as a potential conflict of interest.
